# Special Relativity at the Quantum Scale

**DOI:** 10.1371/journal.pone.0115810

**Published:** 2014-12-22

**Authors:** Pui K. Lam

**Affiliations:** Department of Physics and Astronomy, University of Hawaii at Manoa, Honolulu, Hawaii, United States of America; Shanxi University, China

## Abstract

It has been suggested that the space-time structure as described by the theory of special relativity is a macroscopic manifestation of a more fundamental quantum structure (pre-geometry). Efforts to quantify this idea have come mainly from the area of abstract quantum logic theory. Here we present a preliminary attempt to develop a quantum formulation of special relativity based on a model that retains some geometric attributes. Our model is Feynman's “checker-board” trajectory for a 1-D relativistic free particle. We use this model to guide us in identifying (1) the quantum version of the postulates of special relativity and (2) the appropriate quantum “coordinates”. This model possesses a useful feature that it admits an interpretation both in terms of paths in space-time and in terms of quantum states. Based on the quantum version of the postulates, we derive a transformation rule for velocity. This rule reduces to the Einstein's velocity-addition formula in the macroscopic limit and reveals an interesting aspect of time. The 3-D case, time-dilation effect, and invariant interval are also discussed in term of this new formulation. This is a preliminary investigation; some results are derived, while others are interesting observations at this point.

## Introduction

Einstein [1], Feynman [2], Schwinger [3], and others have pointed out that there are fundamental difficulties and inconsistencies inherent in our present-day quantum theory that treats space-time as a continuum. Wheeler [4] believes that the lattice approach to space-time is not satisfactory and will not lead to a true understanding of the quantum nature of space-time (pre-geometry). An intriguing idea, which is strongly advocated by Weizsacker [5], is that the space-time structure is an outcome of quantum theory. This idea is further emphasized by Wheeler [4], [6], Fingelstein [7]–[9], and many others [10], [11] that the fundamental rule of nature is governed by quantum theory of information (quantum logic), and the apparent space-time structure is inferred only in the macroscopic limit.

Although the field of quantum logic is quite advanced, the axiomatic formulation is rather abstract and often lacks “physical semantic” according to Weizsacker [5]; that is, the mathematical formalism is not given a meaning in physics. In this paper, we will attempt to bridge the gap and present a physical model for quantum kinematics. We will focus on the kinematics aspect of special relativity. Our basic approach is to compare the kinematic “measurements” of a quantum system by two quantum inertial observers; that is, both the system and observers are described by quantum principles. Since there is no definitive theory on quantum measurements, we will have to make some assumptions about the “measurements”.

Before presenting our model, it is useful to review the essential steps by which the theory of special relativity for continuous space-time is formulated [12]:

1. Special relativity is based on two postulates:
  - Postulate 1: The principle of relativity states that all physical laws must be invariant with respect to all inertial reference frames.
  - Postulate 2: The speed of light (*c*) has the same value for observers in different inertial frames [13], [14].
2. The fundamental coordinates for describing events are assumed to be position and time.
3. The Lorentz transformation of coordinates is shown to be consistent with Postulates 1 and 2.

Our new approach is to identify the following (using a specific model as a guide):

1. The quantum version of Postulates 1 and 2.
2. The appropriate quantum “coordinates”.
3. The transformation rules for these quantum “coordinates” that are consistent with the new postulates.

A word of caution is needed. Wheeler has emphasized the importance of not assuming any *a prori* geometric properties when one wants to “derive” the geometric properties of space-time from pre-geometry [4]. This is what Weizsacker called the “semantical consistency” [5]. However, Weizsacker also pointed out that in order to describe a “new” theory to someone (who does not know the theory yet), one must use the “old” language that may not be completely consistent with the “new” theory. During the course of the explanation, one modifies the language, in a bootstrap process, to achieve consistency with the new theory. In our presentation, we will first describe our physical model in the language of continuous space-time, i.e., with all its geometric attributes. Once we identify the essence of the physical model, we will re-cast our quantum version of the postulates in a language that does not make any reference to geometry. Weizsacker also made a conjecture that no theory at the present is fully semantically consistent; perhaps only the “final” theory of everything would satisfy that requirement. We will point out the loose ends in our model.

In the Methods section, we will describe the Feynman “checker-board” model and identify the relevant features that guided us in formulating the quantum postulates for special relativity. In the Results and Discussion section, we will derive the velocity transformation rule for 1-D and present the generalization to 3-D. In addition, we will illustrate how the time-dilation effect can be visualized using Feynman's model and point out an interesting connection between the invariant space-time interval and the area of the x-t region that encloses all allowable paths. In the Conclusions section, we will summarize the key findings and list some loose ends that need further investigations.

## Methods

We will use Feynman's path-integral trajectory for a 1-D relativistic free particle ("checker-board" model) [15] to guide us in formulating the quantum postulates for special relativity.

### Feynman's Checker-Board Model

Feynman originated this model to derive the 1-D Dirac equation from his path-integral formulation of quantum mechanics. Jacobson gave a "spinor-chain" path-integral formulation for the 3-D Dirac equation [16]. Since Feynman's version has a more direct connection with space-time, we will describe his model first. Then, we will re-cast Feynman's model in terms of a spinor-chain description, which may be more closely connected with quantum logic.

**Figure 1:**
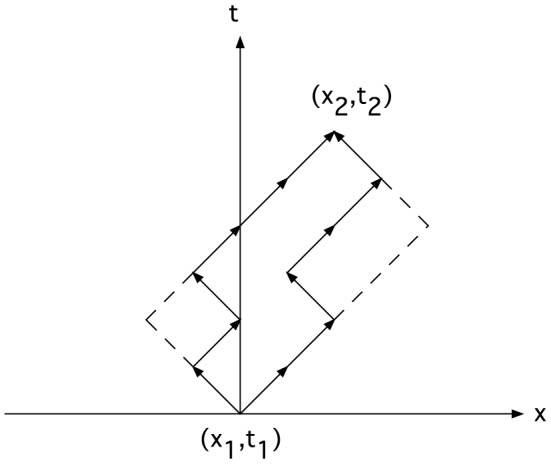
Feynman's “checker-board” model for the trajectories of a 1-D relativistic particle. The paths consist of segments with velocities equal to 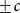. All possible paths are contained inside the dashed rectangle.

In the checker-board model, a particle in 1-D moves forward and backward in space at the speed of light [17]. The trajectory is a zigzag path in the x-t plane (see Fig. 1), hence the name “checker-board”. The amplitude for the particle to move from (x_1_,t_1_) to (x_2_,t_2_) has contributions from all possible paths that lie within the dashed-line rectangle in Fig. 1. (We assume that the particle does not travel backward in time; see remarks in the last section of this paper.) To facilitate the enumeration of paths, the time axis is divided into equal steps of width 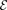. There are 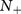 number of positive steps and 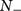 number of negative steps. The continuum limit is obtained by letting 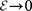 and 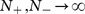. Feynman proposed that the amplitude is given by [15]

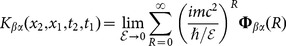
(1)where R is the number of turns in the zigzag path and 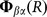 is the number of paths having R turns with 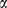 and 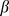 denoting the initial and final step directions, respectively. For example, if the particle leaves x_1_ by taking a forward step and arrives at x_2_ by taking a backward step, then 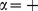 and 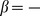. The first factor inside the sum, 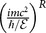, is the quantum amplitude for having R turns. Note that if m = 0, then the amplitude for turning is zero. Therefore, a massless particle moves along a straight path at the speed of light; only particles with finite mass move in zigzag paths in the x-t plane.

It has been shown [18]–[20] that the continuum limit of the propagator in Eq. (1) is equivalent to that derived from the 1-D Dirac equation. Here, we shall focus only on the kinematics implication of this model. The relevant feature of this model to our present discussion is that, at the quantum level, there is only one relative speed, namely the speed of light (*c*). Put more precisely, the eigenvalues of the velocity operator along any direction are 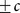.

To make a connection with measurements at the macroscopic level, we define an average velocity
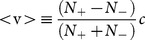
(2)where the bracket sign, <>, is used to remind us that the velocity is an average over the entire path. In the macroscopic limit where 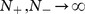, we can identify 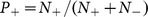 and 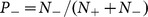 as the probability of occurrence for a positive and a negative step, respectively. Equation (2) can be re-written as
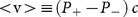
(3)


This definition of the macroscopic velocity does not refer to space explicitly; it only depends on the probability of occurrence for each velocity quantum state: the + state and the - state. We identify the quantum kinematic coordinates to be the number of + steps (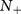) and the number of – steps (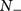), or equivalently, the probabilities 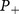 and 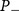. Continuous space-time can be recovered in the limit of 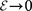 and 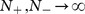,
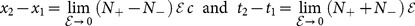
(4)


The + and – step directions are referenced with respect to a given “inertial observer”. We avoid using the term inertial reference *frame*. Instead we will take the viewpoint that kinematics (in fact all physical laws) must be defined in terms of information available to the observer locally. The observer need not be macroscopic; an elementary observer is in fact a quantum object which obeys quantum principles. We consider a free particle obeying Eq. (1) to be an elementary inertial observer. In classical special relativity theory, one considers two inertial observers at relative constant velocity. Here, the two quantum observers do not have a constant relative velocity at the quantum time scale, but rather an average relative velocity as defined by Eq. (3). Note: At present, we do not have a theory of measurement that tells us how the observer obtains the information about the system under observation, so we simply assume that such information is available to the observer. The important requirement is that such information obeys the principle of relativity.

### Quantum Postulates for Special Relativity

We are ready to state the quantum version of Postulates 1 and 2 of special relativity:

- Q-Postulate 1: The principle of relativity remains valid at the quantum level, namely, that all physical laws must be invariant with respect to all quantum inertial observers.
- Q-Postulate 2: At the quantum scale, the eigenvalues of the velocity operator along any direction can only be *+c* or –*c*. (The crucial point is that this speed is finite. By the virtue of Q-Postulate 1, this finite speed must be the same for all inertial observers.)

## Results and Discussion

We will derive the velocity transformation rule for 1-D and present the generalization to 3-D. The transformation rule reveals a very interesting aspect of time. We will illustrate how the time-dilation effect can be visualized using Feynman's model and point out an interesting connection between the invariant space-time interval and the area of the x-t region that encloses all allowable paths.

### Velocity Transformation Rule for 1-D

**Figure 2:**
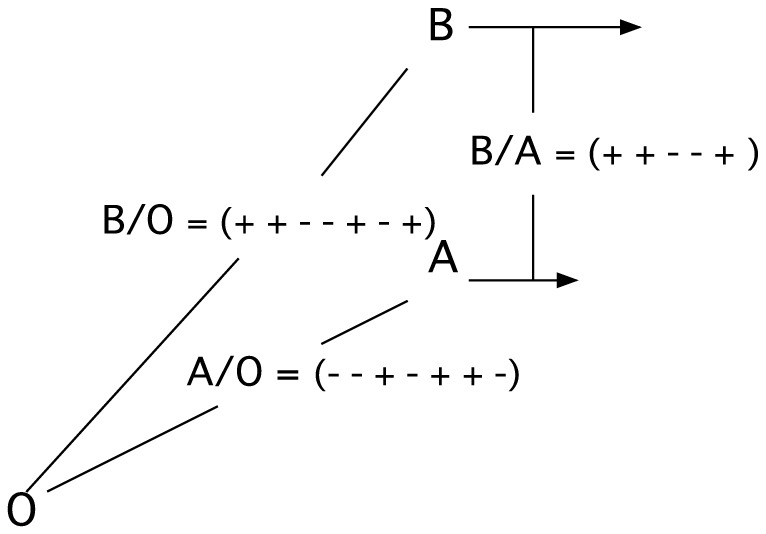
Bit-string representations of relative motions. The motion of B with respect A (B/A) can be deduced from the motions of B with respect to O (B/O) and A with respect to O (A/O) using the rules listed in Table 1.

In our quantum formulation, the probabilities are the fundamental quantities. We will deduce the transformation rule for the probabilities, which we call the P-transformation. The transformation rule must be consistent with Q-Postulate 1 and Q-Postulate 2. Consider three free particles, A, B, and O. Given that A is moving at a relative average velocity <V_A/O_> with respect to O, and that B is moving at a relative average velocity <V_B/O_> with respect to O, we wish to find <V_B/A_>. That is, we need to deduce 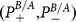, given 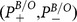 and 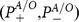. To find the velocity of B with respect to A, we examine the motion of B “as seen by” A. To facilitate the analysis, we use a bit-string representation of the paths (see Fig. 2). Heuristically, the bit-strings can be thought of as the experimental records of the motions of B and A as measured by O. The four possible pairs of motions of B and A as viewed by O are: 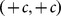, 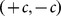, 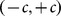, and 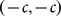. According to Q-Postulate 2, the only symbols available to A for describing the motion of B are 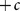 and 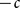. Intuitively, the two processes 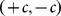 and 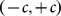 that exhibit relative motions should be assigned 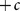 and 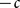, respectively. As for the processes 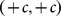 and 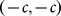, where there are no relative motions, what symbol can be assigned to these events? We cannot assign zero to these two events, because zero is NOT an allowed symbol. We cannot assign 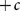 or 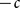 for the following reason. Suppose we assign 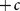 to 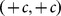. If we interchange B and A, the relative velocity should change sign, hence we are forced to assign 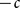 to 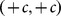, which leads to an ambiguous transformation rule. *The only conclusion is that there is no symbol assigned to* 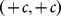 *or* 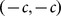. A possible interpretation is that nothing is happening or *no time has elapsed* as far as B and A are concerned, when O observes 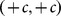 and 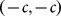. These rules are summarized in Table 1.

**Table 1:** Rule table for determining relative velocity at the quantum scale.

B/O	A/O		B/A
*+c*	*+c*	= >	no symbol
*+c*	*−c*	= >	*+c*
*−c*	*+c*	= >	*−c*
*−c*	*−c*	= >	no symbol

B/O, A/O, and B/A are motions of B as viewed by O, A as viewed by O, and B as viewed by A, respectively.

The normalized probabilities, 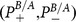, are defined as
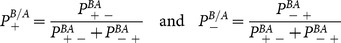
(5)where 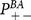 is the joint probability for B taking a positive step while A taking a negative step as viewed by O, while 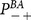 is the joint probability for the reverse directions. In general, the joint probabilities will depend on the pair of paths under consideration; one must compute the ensemble-average over all possible pairs. It is difficult to compute the ensemble-average analytically. In the macroscopic limit where the time of observation, 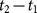, is much longer than the characteristic time for making a turn, 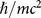, we assume that the ensemble-average joint probability can be approximated by the product of probabilities, i.e.,
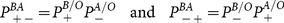
(6)


This is a reasonable assumption because the motions of B and A are independent. We will show that the P-transformation stated in Eqs. (5) and (6) leads to the Einstein's velocity-addition formula. Here are the details:
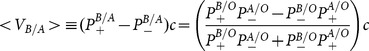
(7)


From the definition of velocity and the normalization of probability, 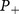 and 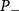 can be expressed in terms of <v>,
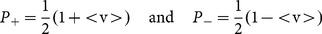
(8)where <v> is expressed in units of *c* to simply the expressions. Therefore,
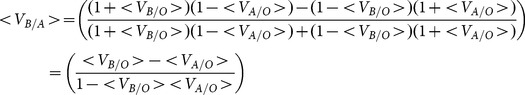
(9)which is the well-known Einstein's velocity-addition formula when <V_B/O_> is parallel to <V_A/O_>.

It is gratifying that such simple rules (Table 1) for combining velocities at the quantum scale lead to the Einstein's velocity-addition formula in the macroscopic limit. We should emphasize that the normalization of probabilities in Eq. (5) is crucial for obtaining the correct denominator in the relativistic velocity-addition formula (the denominator of Eq. (9)). Recall that the normalization came about because we must not count “events” where there is no relative motion between B and A; in fact, as far as B and A are concerned, these events do not exist and no time has elapsed. At the quantum time scale, 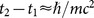, we do not expect that the ensemble-average joint probabilities can be approximated by Eq. (6), and therefore, there may be quantum correction to the velocity-addition formula.

### Generalization to 3-D

The generalization of the checker-board model to 3-D is not simple. As pointed out by Rosen [19], one cannot simply define paths on a cubic lattice in space with two components of velocity equal to zero and the third component equal to 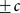 during each step interval. This is because the three components of velocity do not commute in the relativistic limit. Jacobson generalized the idea of velocity-direction change at each corner to quantum-state change in the abstract Hilbert space. He used spinors to represent the states of the particle. Jacobson was successful in obtaining a path-integral representation for the 3-D Dirac equation in terms of these spinor states. Although the space-time picture is not very evident in the spinor representation, the mathematical connection between spinors and space-time have been suggested and explored before [21], [22].

First, we must clarify what we mean by “the only speed at the quantum level is *c*” (Q-Postulate 2). From the solution to the Dirac equation, we know that the velocity operator can be represented by the Pauli spin matrices, and that the eigenvalues of the velocity along any direction are 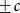. Since the different components of velocity do not commute, there is no simultaneous eigenstate for all three components. At the quantum scale, the precise value of the total velocity (or the total speed) is not known; only one component of the velocity can be known precisely at a time, and that velocity component can only be 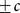.

Now, we generalize our 1-D result to 3-D. The velocity operator (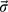), in units of c, is given by
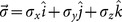
(10)where
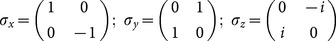
(11)


We use the eigenvectors along the x-direction as the basis, so that the notation is consistent with what we have used in the 1-D case. Note that these are not the conventional definitions of the Pauli matrices. The probabilities 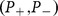 are generalized to a 2×2 density matrix,
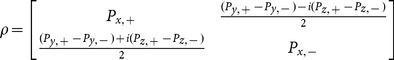
(12)where 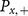 is the probability for a measurement of the velocity along the x-direction would produce the +x eigenstate, and similar definitions for other directions. The probabilities for each direction sum to 1; i.e., 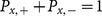, 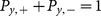, and 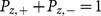. The macroscopic velocity is given by the trace of 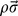,
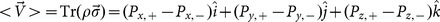
(13)


The probabilities along different directions are NOT independent. We will now show that the average speed is never greater than *c* (i.e. no violation of relativity at the macroscopic scale). Consider the square of the average velocity; it can be expressed in terms of the trace and the determinant of 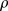,
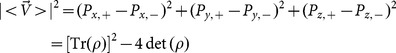
(14)


The trace of 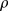 is equal to 1. The determinant can be deduced by finding a basis such that 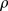 is diagonal. Since the diagonal elements are non-negative (probabilities) and their sum is equal to 1, the determinant of 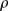 can only vary between 0 and 1/4. Since the trace and the determinant are independent of the basis, Eq. (14) implies that the average speed can never be greater than 1 (in units of *c*). For comparison, the average of the velocity-squared is given by
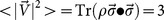
(15).

Since the macroscopic measurement of velocity is given by Eq. (14), not Eq. (15), hence there is no violation of special relativity. Finally, the density matrix can be re-expressed as
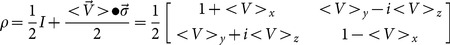
(16)where I is the identity matrix. Although Eq. (16) is a useful expression for 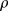, we must remember that 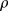 is intrinsically a function of the probabilities.

In regard to the velocity-addition formula, we can pose the problem in the following way. Suppose A and B are moving at 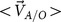 and 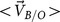 with respect to O, respectively. Given the density matrices, 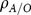 and 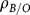, how do we find 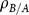 in terms of 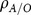 and 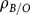
? In the 1-D case, we were able to compare the *+c* and *−c* symbols and deduce the transformation rules; it is not clear how to compare the *+c* and *−c* symbols for different directions. For the case where 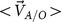 and 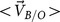 are parallel (equivalent to 1-D), we empirically arrive at the following generalization,
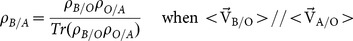
(17)where 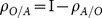 as evident from Eq. (12) or Eq. (16).

For the general 3-D case, our empirical result is
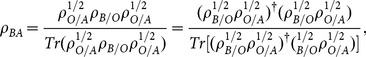
(18)where 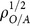 is defined by 
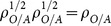
 and is the positive root of 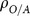. The second expression in Eq. (18) explicitly shows the Hermitian property of the density matrix. One may be tempted to identify
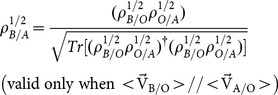
(19)


However, this quantity is not Hermitian unless 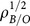 and 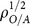 commute. The special case of Eq. (17) (when 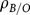 and 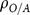 can be diagonalized by the same basis) is included in Eq. (18).

We will illustrate Eq. (18) for the case where 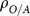 is diagonal (i.e., choosing 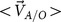 to be along the x-direction). For this case, we have
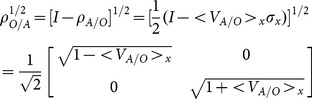
(20)and
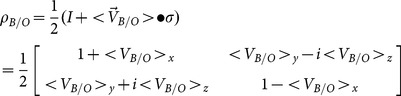
(21)


Hence,
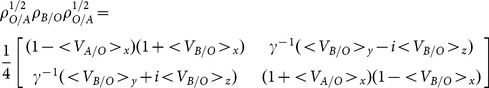
(22)where 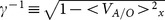 and
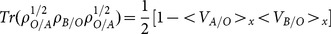
(23)


Therefore,
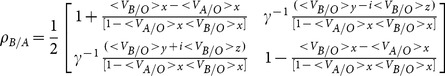
(24)


Comparing Eq. (24) with Eq. (16), one can read off the components of the velocity 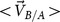, and these components agree with Einstein's velocity-addition formula.

Note: Unlike the 1-D case, where we are able to deduce the rules for combining velocities that are valid at the quantum time scale (Table 1), for the 3-D case we are only able to state an empirical formula for velocity-addition that is valid in the macroscopic limit.

### Clock and Time-Dilation Effect

**Figure 3:**
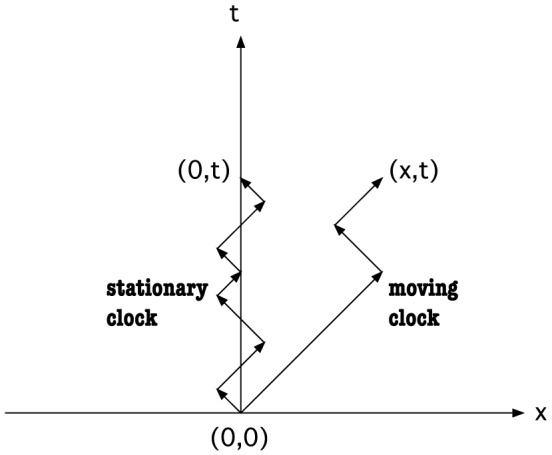
Typical path for a stationary clock and that of a moving clock as viewed by an observer. Each directional change is a “tick” of the clock. The time-dilation effect is illustrated very clearly here by the moving clock changing its direction less frequently than the stationary clock.

A clock signifies the passage of time by a change of its (quantum) state. The motion of a quantum “free” particle could, in principle, be used as a clock; every change of direction signifies a “tick” of this clock. In the absence of other clocks, the interval between ticks will be perceived to be equal. Using the motion of a quantum free particle as a clock, we can illustrate the time-dilation effect. We compare the number of ticks from a “moving” clock with that from a “stationary” clock as viewed by the same observer O. The motions of these two clocks as viewed by O are depicted in Fig. 3. The moving clock changes its direction less frequently because it takes unequal numbers of positive and negative steps. Hence, the moving clock appears to run slower than the stationary clock as far as observer O is concerned. In fact, if the velocity of the moving clock is *c*, then there will be no change of direction at all and there will be a complete time dilation. To quantify the above description, we compute the typical number of turns, R*, for the stationary and moving clocks. We can evaluate the most probable number of turns by finding the largest absolute value of the summand in Eq. (1). Following Jacobson and Schulman [18], the result is
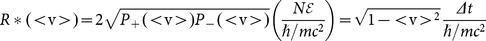
(25)


The moving clock ticks at a slower rate compared to the stationary clock as given by the ratio
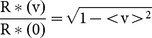
(26)


We should clarify the interpretation of this result: it states that, with respect to the same inertial observer, a stationary clock ticks more frequently than a moving clock over the same duration 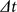 of the observer's own clock. However, if two inertial observers in relative motion are observing the same clock then the number of ticks is invariant because Eq. (25) indicates that the number of ticks is proportional to the clock's own proper time interval 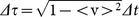.

### Invariant Space-Time Interval

**Figure 4:**
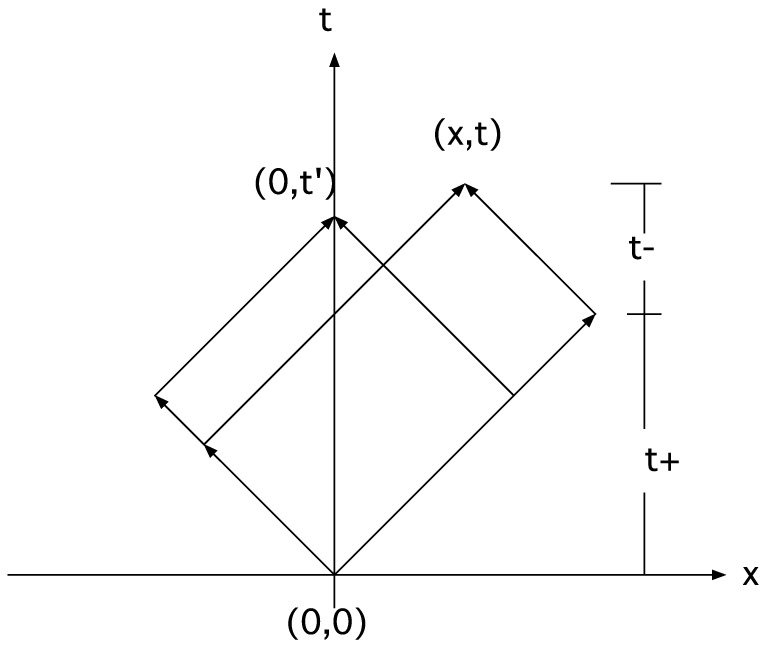
Region in x–t plane enclosing all possible quantum paths between two events as viewed in two reference frames. The area of this region is proportional to the invariant space-time interval; therefore, its numerical value is independent of reference frames.

We have noticed an interesting connection between the space-time interval, 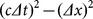, and the area of the rectangle in the x–t plane that bounds all possible paths connecting the initial and final events. Let the coordinates for these two events be (0,0) and (x,t) in the unprimed frame and (0,0) and (x',t') in the primed frame. In these two frames, the possible paths connecting these two events are bounded inside their corresponding rectangles in the x–t plane (see Fig. 4). The area of the rectangle in the unprimed frame is given by
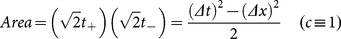
(27)where we have used the fact that 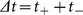 and 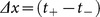. Hence, the invariance of the area enclosing the paths would imply the invariance of the space-time interval. Now, the question is: Does the invariance of the area follow from some fundamental principle? An initial guess may be that the area is proportional to the total number of paths connecting the two events; hence, one could propose a principle that the number of paths connecting two events is invariant of frames of reference. This sounds plausible. However, a calculation of the total number of paths seems to invalidate this presumption. The total number of paths (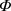) is given by
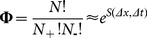
(28)where
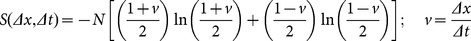
(29)


The number of time steps, 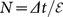, is an invariant because both 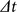 and 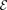 should transform alike. It is evident from Eq. (29) that the total number of paths is not a relativistic invariant. Our preliminary analysis indicates that one must take into account that not all paths have the same probability. If we consider only paths that contribute significantly to the amplitude in Eq. (1), then the number of significant paths is proportional to the invariant space-time interval. This raises an issue that the dynamical aspect of a free particle (the amplitude factor in Eq. (1)) is connected to the kinematical aspect. Such potential connection needs further investigation.

## Conclusions

We conducted a preliminary attempt to develop a quantum formulation of special relativity based on Feynman's checker-board model for motion of a free particle at the quantum scale. Feynman's model was chosen because of its success in leading to the well-known Dirac equation. An intriguing feature of Feynman's model is that particles move at only one speed, namely the speed of light, at the quantum scale. We formulated the quantum version of the postulates for special relativity and derived the rules for determining relative velocity at the quantum scale. These simple rules lead to Einstein's velocity-addition formula in the macroscopic limit. At the quantum time scale, 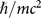, there may be quantum correction to Einstein's velocity-addition formula. We discovered an interesting aspect of time. It is possible to interpret that when two particles have no relative motion, there is no elapse of time as far as those two particles are concerned. Typically, one thinks of time simply marching on, independent of one's motion. This interesting aspect of time is worth further investigation. Using the motion of a free particle as a clock, we provided a simple demonstration of the time-dilation effect.

An important aspect of the present formulation is that it makes no explicit reference to space (or its geometric properties). For example, the definition of velocity in Eq. (3) requires only the existence of velocity eigenstates and a definition for the probability of occurring in each velocity eigenstate. This approach is in line with Wheeler's idea of pre-geometry.

There are few loose ends that we should mention:

1. We have considered only paths that move forward in time. Paths that are allowed to move backward in time have been discussed in a paper by Ord [23] in connection with quantum interference arising from charge conservation. At present, we are uncertain how to deal with this type of path in determining relative velocity.
2. We made an observation that the area of the rectangle in the x–t plane enclosing all the possible paths is proportional to the space-time interval (Eq. (27)). The total number of paths within the rectangle is not proportional to the space-time interval. However, the number of significant paths is proportional to the space-time interval. Significant paths are those maximize the summand in Eq. (1). The potential connection between the dynamical aspect and kinematical aspect of motion needs further investigation.
3. Unlike the 1-D case, we are unable to directly derive the velocity transformation rule for the 3-D case from the postulates. Basically, by “reverse engineering”, we arrived at an empirical formula (Eq. (18)) that gives the correct result in the macroscopic limit.

We have presented some preliminary ideas on how relativistic kinematics may be viewed at the quantum scale. Much more work is needed to find a complete self-consistent formulation. We hope that these preliminary results will inspire others to pursue further investigations.
